# Long-Term Study of Physical, Haematological, and Biochemical Parameters in Cattle with Different Embryo Origins

**DOI:** 10.3390/ani15121763

**Published:** 2025-06-14

**Authors:** María Serrano-Albal, Jon Romero-Aguirregomezcorta, Sebastián Cánovas, Sonia Heras, Joaquín Gadea, Pilar Coy, Raquel Romar

**Affiliations:** 1 Department of Physiology, Faculty of Veterinary, University of Murcia, 30100 Murcia, Spain; maria.serranoa@um.es (M.S.-A.); jon.romero@um.es (J.R.-A.); scber@um.es (S.C.); sonia.heras@um.es (S.H.); jgadea@um.es (J.G.); pcoy@um.es (P.C.); 2 Biomedical Research Institute of Murcia Pascual Parrilla–IMIB, 30120 Murcia, Spain

**Keywords:** cattle, assisted reproductive technologies, haematological profile, biochemical profile, long-term study

## Abstract

Assisted reproductive technologies are vital in cattle breeding to improve genetic selection and productivity. While early-life differences between artificially inseminated (AI) and in vitro-produced (IVP) cattle have been studied, long-term physiological, haematological, and biochemical effects remain unclear. This observational study assessed AI and IVP cattle from 1.5 to 5 years of age to determine if early differences persist. IVP cattle were produced after the transfer of the embryo produced by supplementing (RF-IVP group) or not supplementing (C-IVP) the embryo culture with oviductal and uterine fluids. Physical evaluations showed body mass index increased until 3.5 years, while temperature and respiratory rate declined with age, with no significant differences between AI and IVP groups. Haematological analysis revealed age-related changes, including decreased red and white blood cell counts and increased mean corpuscular volume and haemoglobin. AI cattle had higher white blood cell counts than IVP groups. Sex significantly influenced many haematological variables. Biochemical analysis showed age-related increases in total protein, creatinine, and urea, and decreases in glucose and alkaline phosphatase. AI cattle had lower cholesterol and creatinine than IVP groups. Despite group differences, all values remained within normal ranges. Sex affected albumin, cholesterol, triglycerides, and creatine kinase. This study provides the first long-term haematological and biochemical reference values for cattle from different reproductive methods, showing that age is the main influencing factor and supporting IVP cattle as a viable alternative to AI in breeding programs.

## Simple Summary

This study looked at whether cows born through different assisted reproductive methods grow and stay healthy over time. Some cows were born using artificial insemination, while others were produced in the lab and implanted into mother cows. The researchers followed these animals from one and a half to five years of age, measuring their weight, temperature, heart, and breathing rates, and analysing their blood and chemical levels. While some small differences were found depending on how the animals were conceived, such as slightly lower cholesterol or white blood cells, most health indicators stayed within the normal range for all animals. The biggest changes were linked to age, not to the way the cows were born. For example, as the cows got older, their weight increased, their body temperature and breathing slowed down, and some blood and biochemical levels changed naturally. The study found that both lab-produced and traditionally conceived cows developed similarly over the years, showing that both methods are safe and lead to healthy animals. These results are important for farmers and scientists, as they confirm that newer reproductive technologies can be used confidently to breed healthy livestock for food production and farming.

## 1. Introduction

The increased demand for beef and dairy products has made assisted reproductive technologies (ART) essential for breeding [1]. In the industry, ART allow for shorter generation intervals and the transfer of genetically beneficial traits to livestock in a highly advantageous, cost-effective, and biosecure manner [2]. Artificial insemination (AI) has been widely used for decades and is the standard reproductive method in the industry in both intensive and extensive systems [3]. However, the use of in vitro embryo production followed by embryo transfer (IVP-ET) has also become increasingly popular and the refinement of IVP methods has allowed for more than 1 million embryo transfers in 2022 [4].

Several long-term studies, mostly in humans, have reported health effects of ART [5], with IVP children showing higher blood pressure or metabolic problems [6,7]. However, there is a large body of literature showing no effect of ART or transient effects that resolve in adulthood [8,9]. In cattle, studies comparing IVP and AI-derived animals have mostly focused on pre-implantation differences, such as embryo number and quality, ploidy or epigenetics [10,11,12], and pregnancy and birth rates [13,14]. Very few studies have focused on physical and blood parameters, and these often only include data from the first days or weeks of the animal’s life [15,16,17]. However, due to the long life expectancy of cattle, especially dairy breeds, longer term studies are needed. There are a few studies that have collected information up to the first year of life [18,19,20,21]. but there is very little information on older animals, and usually only including differences between breeds but not between reproduction methods or age [21].

Physiological, haematological, and biochemical parameters are critical indicators of health, metabolic efficiency, and long-term viability in cattle. Physiological metrics such as body mass index (BMI), temperature, and respiratory rate reflect developmental progress and adaptive capacity, while haematological profiles (e.g., red/white blood cell counts and haemoglobin levels) provide insights into immune function and oxygen transport efficiency. Biochemical markers such as cholesterol, creatinine, and alkaline phosphatase levels are essential for evaluating organ function and metabolic stress. In previous studies, we have reported physiological, haematological, and biochemical differences between cattle produced by AI and different IVP protocols [18,22] Specifically, during the first year of life, evident differences appear between calves derived from standard embryo production and AI [18]. Briefly, the embryonic origin of animals affected body mass index, body temperature, respiratory rate, basophils count, withers, reticulocyte haemoglobin content, globulin concentration, creatinine concentration, and glucose concentration [18] This study aims to investigate whether these variations between animals of different embryo origins remain the same, vary, or resolve with age by studying the same herd up to 5 years of age.

## 2. Materials and Methods

### 2.1. Animals

Cattle were born in the period between August 2018 and May 2019, at a commercial farm (El Barranquillo SL, Torre Pacheco, Murcia, Spain) [14] and six days after birth, they were transported to the Veterinary Teaching Farm (VTF) at the University of Murcia (Spain). Animals were born after artificial insemination and after the transfer of in vitro embryos (IVP) produced in two different systems using slaughterhouse ovaries (crossbred Charolais and/or Limousin) and gestated by Holstein recipient cows [14]. So, the three experimental groups are (i) animals born after the artificial insemination of synchronized Holstein cows (AI group); (ii) animals born after the transfer of IVP embryos cultured in a standard system using bovine serum albumin (C-IVP group); and (iii) animals born after the transfer of IVP embryos cultured in a system with reproductive fluids in the culture media (RF-IVP group). All animals were fathered by the same bull (Asturiana de los Valles breed) and followed routinely to perform physical examination and blood collection [14]. Data collected from birth until first year of life are compiled in Lopes et al. [18].

For the current study, animals were evaluated every 6 months from 1 year old until 5 years age. During examinations, each animal was individually kept in a cattle crush. The animal was left to relax for 5–10 min before starting the examination, with blood extraction performed last to reduce stress in the animal. The chosen time points were 1.5, 2, 2.5, 3, 3.5, 4, 4.5, and 5 years old. The herd included seven calves in the AI group (5 males and 2 females), seven calves in the C-IVP group (4 males and 3 females) and four animals in the RF-IVP group (3 males and 1 female). Due to the reduced number of animals at late adulthood, data collected at 4, 4.5, and 5 years old were grouped in ≥4 years group.

No cow calved during the study and animals were enrolled in a health maintenance program managed by the veterinarians at the VTF. Females and males lived in separated pens, but they were kept and fed under the same conditions. All of the animals were housed in open-air yards with roofs and ad libitum access to water and were fed with a grain mix and straw (15.5% crude protein, 2.10% crude fat, 9.30% crude fibre, 7.90% crude ash, 1.40% calcium, 0.51% phosphorus, and 0.41% sodium).

### 2.2. Physical Evaluation

Body weight (kg) was measured with a weight scale (BR 15, Baxtran, Giropes SL, Girona, Spain), and body length (cm, distance from the head to the base of the tail measured with a zoometric measuring tape, Kamer; Ukal, Eschbach, France) and height at withers (cm) were routinely evaluated to determine the animal’s BMI [(body weight/height at withers/body length)/10], calculated according to Tanaka et al. [23].

Furthermore, body temperature (°C, rectal temperature measured with a clinical digital thermometer), heart rate (beats per minute, assessed with a stethoscope), and respiratory rate (RPM, the number of respiratory cycles per minute, based on flank movements) were also included in the evaluations.

### 2.3. Blood Collection and Analysis

Blood samples were collected from the tail using lithium heparin vacutainer tubes (BD Vacutainer, BD Spain, Fraga, Spain) and preserved at 4 °C. Haematological analysis was performed withing 4 h of collection in whole blood using a haematology analyser (Siemens ADVIA^®^ 120, Mountain View, CA, USA) at Interlab-UMU (University of Murcia. Spain). Parameters included in the haematological profile were haematocrit (%), haemoglobin (g/dL), red blood cells (RBC; ×10^6^ cells/μL), mean corpuscular volume (MCV; fL), mean corpuscular haemoglobin (MCH; g/dL), mean cell haemoglobin concentration (MCHC; g/dL), cell haemoglobin concentration mean (CHCM; g/dL), red blood cell distribution width (RDW; %), cell haemoglobin distribution width (CHDW; pg), haemoglobin distribution width (HDW; g/dL); white blood cells (WBC; ×10^3^ cells/μL), neutrophils (×10^3^ cells/μL), lymphocytes (×10^3^ cells/μL), monocytes (×10^3^ cells/μL), eosinophils (×10^3^ cells/μL), basophils (×103 cells/μL), platelets (×10^3^ cells/μL), mean platelet volume (MPV; fL), plateletcrit (PCT; %), platelet distribution width (PDW; %), mean platelet component (MPC; g/dL), mean platelet mass (MPM; pg), large platelets (Large PLT; ×10^3^ cells/μL), reticulocyte haemoglobin content (CHr; pg), reticulocytes (×10^6^ cells/μL), and the mean corpuscular volume of reticulocytes (MCVr; fL).

Biochemical analyses were performed on plasma using an automatic chemistry analyser (Olympus AU400, Tokyo, Japan), after serial dilutions. The inter- and intraassay precision of the methods were linear and below 15%. Plasma was obtained by the centrifugation of blood at 1000 g for 10 min, and then stored at −80 °C until the analysis. The biochemical parameters included were total protein (TP; g/dL; OSR6132), albumin (ALB; g/dL; OSR6102), globulin (GLOB; g/dL; calculated from TP-ALB), creatinine (CREA; mg/dL; OSR6178), urea (UREA; mg/dL; OSR6134), glucose (GLUC; mg/dL; OSR6121), cholesterol (CHOL; mg/dL; OSR6116), triglycerides (TRIG; mg/dL; OSR6118), amylase (AMS; UI/L; OSR6182), lipase (LIP; UI/L; OSR6130), creatinine kinase (CK, UI/L; OSR6178), alkaline phosphatase (ALP; UI/L; OSR6104), gamma-glutamyl transpeptidase (GGT; UI/L; OSR6020), aspartate aminotransferase (AST; UI/L; OSR6109), alanine aminotransferase (ALT; UI/L; OSR6107), and total bilirubin (TB; mg/dL; OSR6112).

### 2.4. Statistical Analysis

Statistical analysis was conducted using SPSS for Windows (Version 28, IBM, Chicago, IL, USA). The data were analysed using a mixed-effects model (when there were missing values) or repeated-measures ANOVA followed by Tukey’s multiple comparison test. Data normality was assessed by the Shapiro–Wilk test. When the data were normally distributed, they were analysed using ANOVA. Data that did not follow a normal distribution were analysed using the Kruskal–Wallis test and Games–Howell test. Regardless of the statistical method used, sex, age, and group were considered fixed effects and a *p*-value < 0.05 was considered significant. Correlation between BMI and biochemical parameters was assessed using a Pearson correlation with a significance level of *p* < 0.05. All data are presented as mean ± standard error of the mean (SEM).

## 3. Results

Mean, maximum, and minimum levels for physical parameters, and haematology and biochemistry analysis at all ages, are detailed in Supplementary Tables S1–S6.

### 3.1. Physical Findings

**Figure 1:**
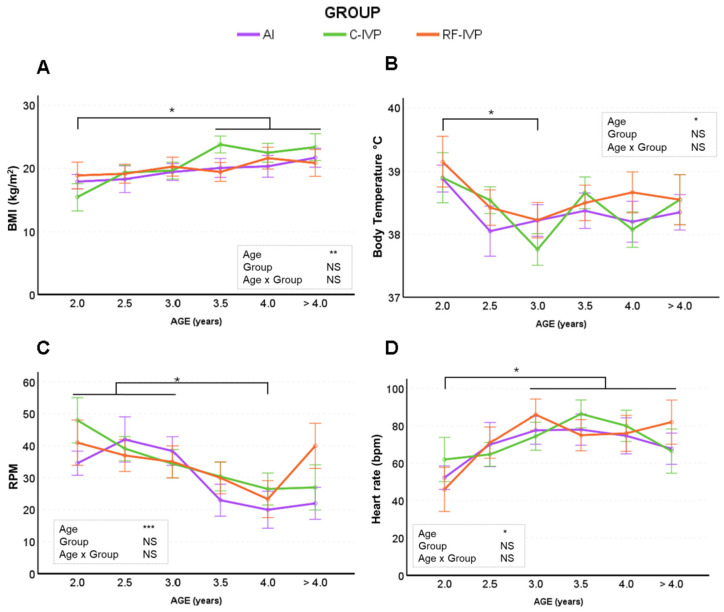
Evolution of physical parameters across age in cattle born by artificial insemination (AI) and transfer of in vitro embryo produced under a standard protocol (C-IVP), and including reproductive fluids (RF-IVP). Data are expressed as mean ± SEM. (**A**) Body mass index (BMI), (**B**) body temperature, (**C**) respiratory rate (RPM, respirations per minute), and (**D**) heart rate (beats per minute). Asterisk shows statistical difference at * *p* < 0.05, ** *p* < 0.01, and *** *p* < 0.001; NS, not significant. Horizontal lines denote that all encompassed time points are significantly different. When the vertical lines are added, only the marked time points are significantly different.

None of the physical parameters evaluated were influenced by the group, but all of the variables were altered by age (Figure 1). Body mass index (BMI; Figure 1A) increased steadily until 3.5 years of age. Body temperature (Figure 1B) decreased by about 1 °C from 2 to 3 years of age and then stabilized until the end of the study at >4 years of age. Respiratory rate (RPM) decreased significantly at 4 years of age compared to previous years (Figure 1C). In contrast, the heart rate (Figure 1D) significantly increased with age. Mean heart rate was higher in females (80.40 ± 3.46 beats per minute) than males (66.75 ± 2.22 beats per minute) (*p*-value =0.008).

### 3.2. Haematological Findings

**Figure 2:**
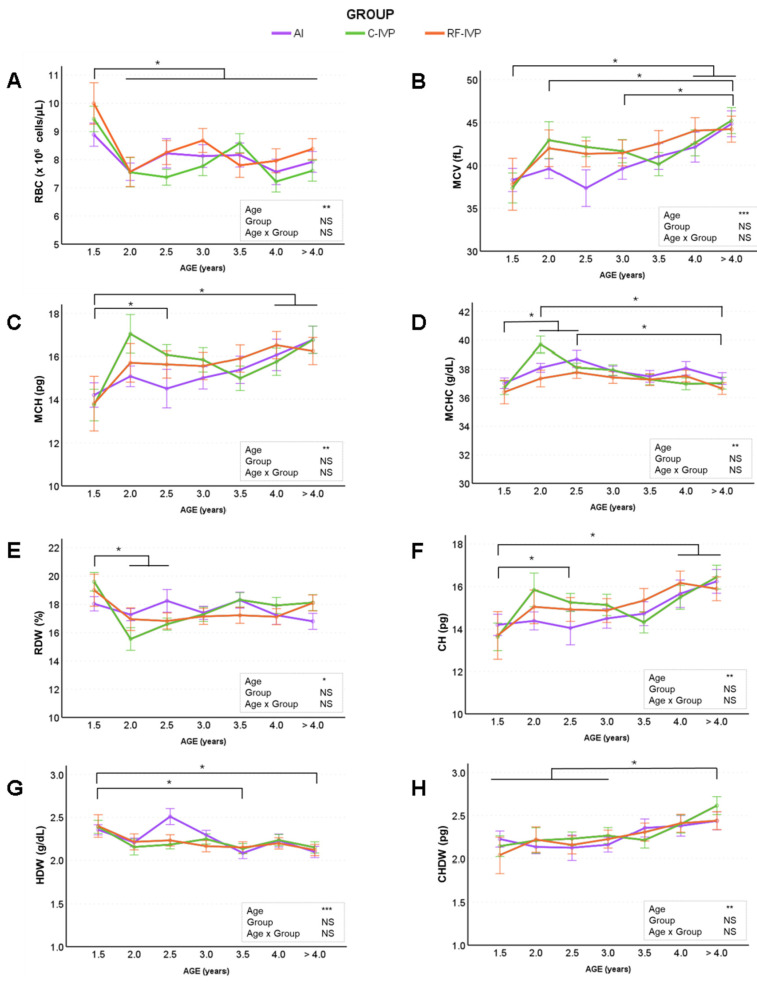
Evolution of haematological parameters (red blood cells) across age obtained in cattle born by artificial insemination (AI) and transfer of in vitro embryo produced under a standard protocol (C-IVP), and including reproductive fluids (RF-IVP). Data are expressed as mean ± SEM. (**A**) Red blood cells (RBC; ×10^6^ cells/μL), (**B**) mean corpuscular volume (MCV; fL), (**C**) mean corpuscular haemoglobin (MCH; pg), (**D**) mean corpuscular haemoglobin concentration (MCHC; g/dL), (**E**) red blood cell distribution width (RDW, %), (**F**) haemoglobin content (CH; pg), (**G**) haemoglobin distribution width (HDW; g/dL), and (**H**) cell haemoglobin distribution width (CHDW; pg). Asterisk shows statistical difference at * *p* < 0.05, ** *p* < 0.01, and *** *p* < 0.001; NS, not significant. Horizontal lines denote that all encompassed time points are significantly different. When the vertical lines are added, only the marked time points are significantly different.

None of the erythrocyte parameters evaluated were influenced by the group. Haematocrit, haemoglobin, and cell haemoglobin concentration mean (CHCM) were not affected by age or group, with mean values of 33.16 ± 0.46%, 12.47 ± 0.18 g/dL, and 36.39 ± 0.08 g/dL, respectively (Supplementary Table S2). However, the remaining erythrocyte variables were influenced by age (Figure 2). The number of erythrocytes (RBC) (Figure 2A) decreased from 1.5 to 2 years of age and remained unchanged for the remainder of the study period, whereas mean corpuscular volume (MCV, Figure 2B) and mean corpuscular haemoglobin (MCH, Figure 2C) increased with age. Mean cell haemoglobin concentration (MCHC, Figure 2D) increased until 2.5 years of age and then began a steady decline with a significant decrease in cattle over 4 years age. In contrast, red blood cell distribution width (RDW, Figure 2E) decreased during the first years of life and then increased slightly until the end of the study. Haemoglobin content (CH, Figure 2F) showed a permanent increase with age, whereas haemoglobin distribution width (HDW, Figure 2G) decreased with age. Finally, values for cell haemoglobin distribution width (CHWD, Figure 2H) were higher in old (>4 years) than in young cattle (1.5–3 years). In summary, MCV, MCH, CH, and CHDW increased with age, whereas RBC, MCHC, RDW, and HDW decreased. Significant differences between males and females were observed in haematocrit, haemoglobin, MCV, MCH, MCHC, CH, CHDW, and HDW, with mean higher values observed in females than males. No age–sex interaction was observed in these variables.

**Figure 3:**
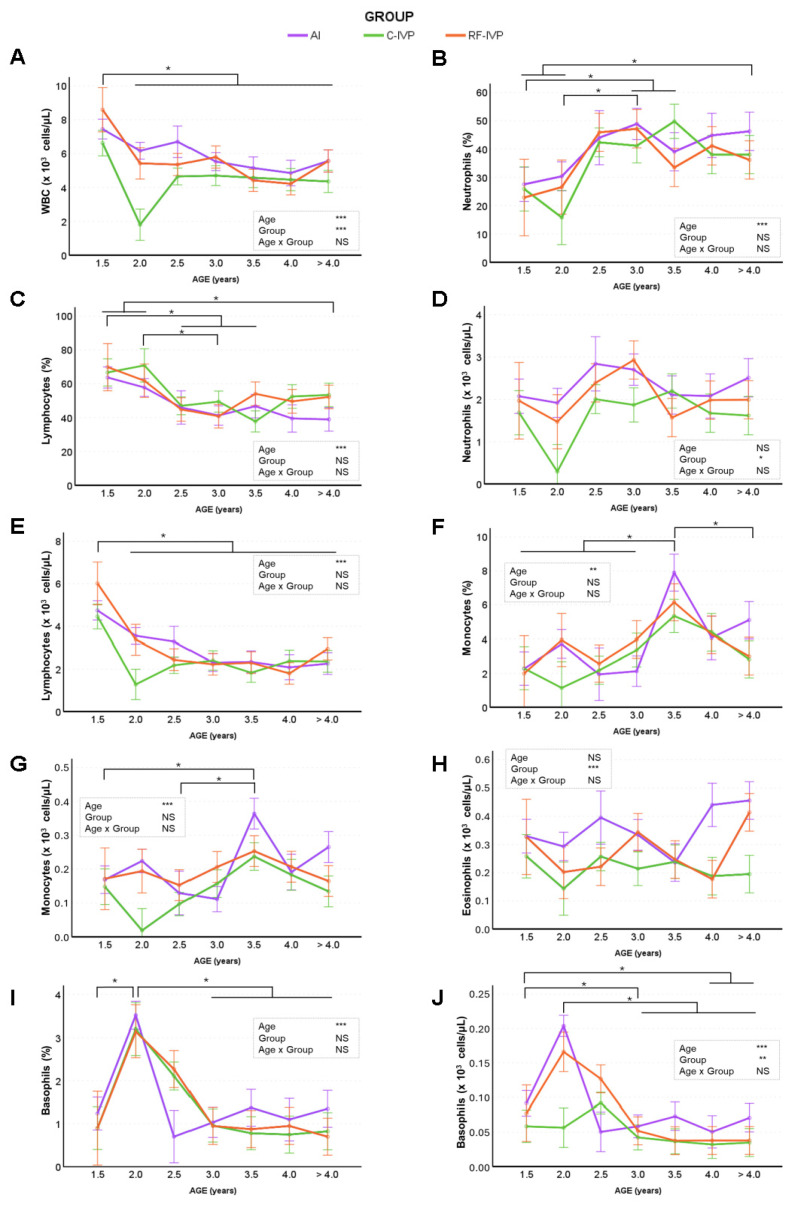
Evolution of haematological parameters (white blood cells) across age obtained in cattle born by artificial insemination (AI) and transfer of in vitro embryo produced under a standard protocol (C-IVP), and including reproductive fluids (RF-IVP). Data are expressed as mean ± SEM. (**A**) White blood cells (WBC; ×10^3^; cells/μL), (**B**) neutrophils (%), (**C**) lymphocytes (%), (**D**) neutrophils (×10^3^ cells/μL), (**E**) lymphocytes (×10^3^ cells/μL), (**F**) monocytes (%), (**G**) monocytes (×10^3^ cells/μL), (**H**) eosinophils (×10^3^ cells/μL), (**I**) basophils (%), and (**J**) basophils (×10^3^ cells/μL). Asterisk shows statistical difference at * *p* < 0.05, ** *p* < 0.01, and *** *p* < 0.001; NS, not significant. Horizontal lines denote that all encompassed time points are significantly different. When the vertical lines are added, only the marked time points are significantly different.

Regarding leukocytes (Figure 3), aging affected all parameters except neutrophil and eosinophil counts, whereas differences between groups were observed for white blood cell (WBC), neutrophil, eosinophil, and basophil counts. Practically throughout life, WBC (Figure 3A) were higher in the AI than in the C-IVP group, with no differences between the IVP groups. The proportion of neutrophils increased in all groups until approximately 3 years of age (Figure 3B), with the lowest number of neutrophils recorded in the C-IVP group compared to the RF-IVP and AI groups (Figure 3D). Lymphocyte percentage (Figure 3C) and concentration (Figure 3E) decreased with animal age, whereas both monocyte percentage (Figure 3F) and concentration (Figure 3G) increased with age. The concentration of eosinophils (Figure 3H) and basophils (Figure 3J) was higher in the AI cattle than in the IVP cattle for practically the whole life span. The proportion of basophils fluctuated up and down at younger ages and then stabilized at older ages (Figure 3I). Among the leukocyte parameters, only the percentage of eosinophils was not altered by age or group. In conclusion, WBC concentrations were found to be higher in AI animals than in C-IVP, which was the standard IVP method. In addition, WBC and lymphocytes decreased with age, neutrophils and monocytes increased with age, basophils fluctuated up and down, and eosinophils were not affected by age. Significant differences between males and females were observed in WBC, lymphocyte number, lymphocyte rate, monocyte rate and eosinophil rate, with mean higher values observed in females than males. No age–sex interaction was observed in these variables.

**Figure 4:**
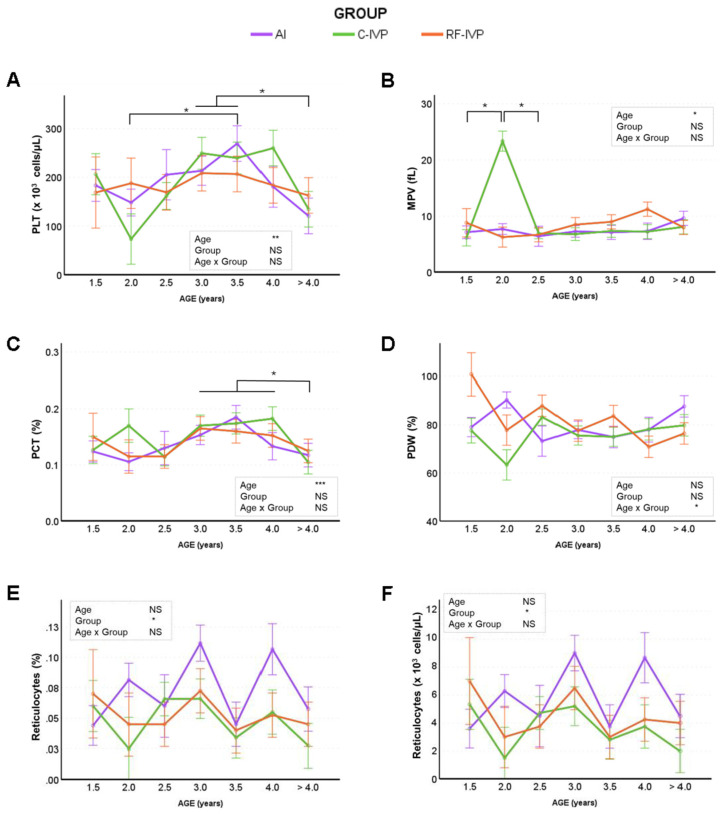
Haematological parameters (platelets and reticulocytes) across age obtained in cattle born by artificial insemination (AI) and transfer of in vitro embryo produced under a standard protocol (C-IVP), and including reproductive fluids (RF-IVP). Data are expressed as mean ± SEM. (**A**) Platelets (PLT; ×10^3^; cells/μL), (**B**) mean platelet volume (MPV; fL), (**C**) plateletcrit (PCT; %), (**D**) platelet distribution width (PDW; %), (**E**) reticulocytes (%), and (**F**) reticulocytes (×10^3^ cells/μL). Asterisk shows statistical difference at * *p* < 0.05, ** *p* < 0.01, and *** *p* < 0.001; NS, not significant. Horizontal lines denote that all encompassed time points are significantly different. When the vertical lines are added, only the marked time points are significantly different.

The results showed that several platelet and reticulocyte parameters were affected by age, while others remained unaffected. The platelet count (PLT, Figure 4A) showed remarkable fluctuations at different ages. At 2 years of age, PLT was relatively low, but increased significantly at 3 and 3.5 years, before decreasing at >4 years of age. Mean platelet volume (MPV; Figure 4B) peaked at 2 years in the C-IVP group (23.35 fL), which was significantly higher compared to RF-IVP (6.25 fL) and AI (7.69 fL). Plateletcrit (PCT; Figure 4C) showed a progressive increase from 1.5 to 3.5 years, followed by a significant decrease at >4 years. Platelet distribution width (PDW; Figure 4D) was influenced by the interaction of age and group. At 1.5 years, RF-IVP had the highest PDW compared to AI and C-IVP, which showed no significant differences. At 2 years, the PDW was highest in the AI group, followed by RF-IVP and C-IVP. After 2.5 years, there were no significant differences in PDW between groups or between ages. Reticulocyte parameters were also affected by group. The AI group showed the highest reticulocyte percentage (Figure 4E) and concentration (Figure 4F) compared to the C-IVP group, while RF-IVP showed intermediate values without significant differences from the other groups. In contrast, several variables were not affected by age or group, including mean platelet content (MPC), platelet distribution width (PCDW), mean platelet mass (MPM), platelet mass distribution width (PMDW), the proportion of large platelets, reticulocyte haemoglobin content (CHr), and mean corpuscular volume (MCV) (mean values shown in Supplementary Table S4). Mean higher values for PCDW were observed in males, and higher CHr and MCVr values in females. No age–sex interaction was observed in these variables.

### 3.3. Biochemical Findings

The correlation study between BMI and biochemical parameters revealed a positive relation between BMI and creatinine only in the AI group (r = 0.584; *p*-value = 0.002), but not in the IVP groups.

**Figure 5:**
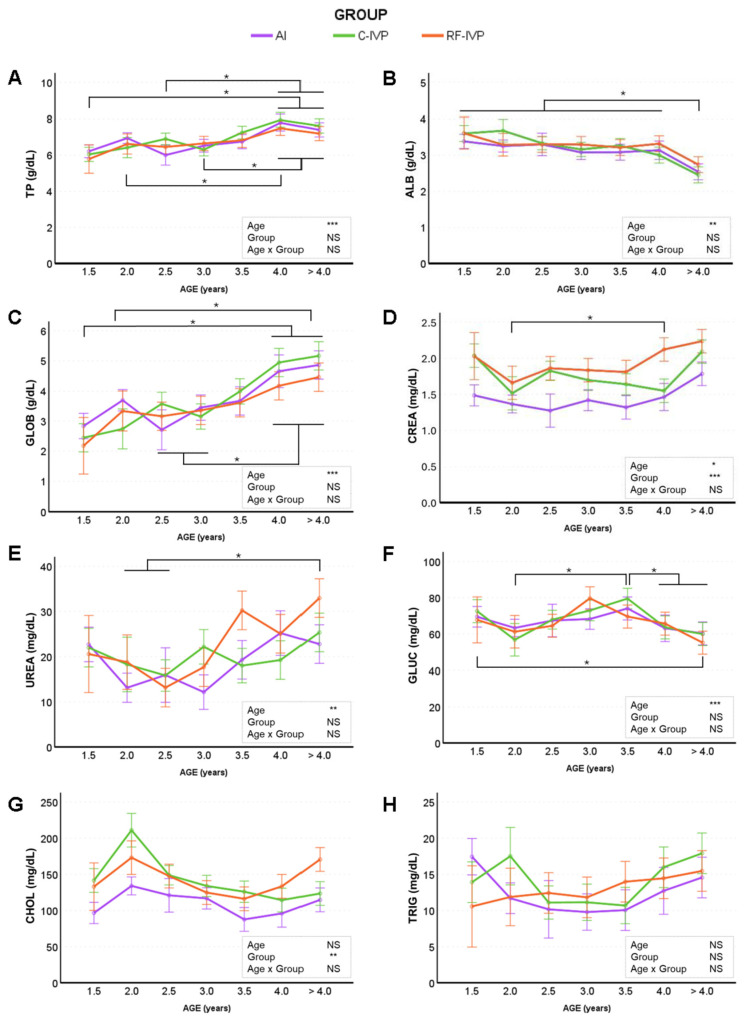
Biochemical parameters (protein, glycaemic and kidney biomarkers) across age obtained in cattle born by artificial insemination (AI) and transfer of in vitro embryo produced under a standard protocol (C-IVP), and including reproductive fluids (RF-IVP). Data are expressed as mean ± SEM. (**A**) Total proteins (TP; g/dL), (**B**) albumin (ALB; g/dL), (**C**) globulin (GLOB; g/dL), (**D**) creatinine (CREA; mg/dL), (**E**) urea (mg/dL), (**F**) glucose (GLUC; mg/dL), (**G**) cholesterol (CHOL; mg/dL), and (**H**) triglycerides (TRIG; mg/dL). Asterisk shows statistical difference at * *p* < 0.05, ** *p* < 0.01, and *** *p* < 0.001; NS, not significant. Horizontal lines denote that all encompassed time points are significantly different. However, when the vertical lines are added, only the marked time points are significantly different.

**Figure 6:**
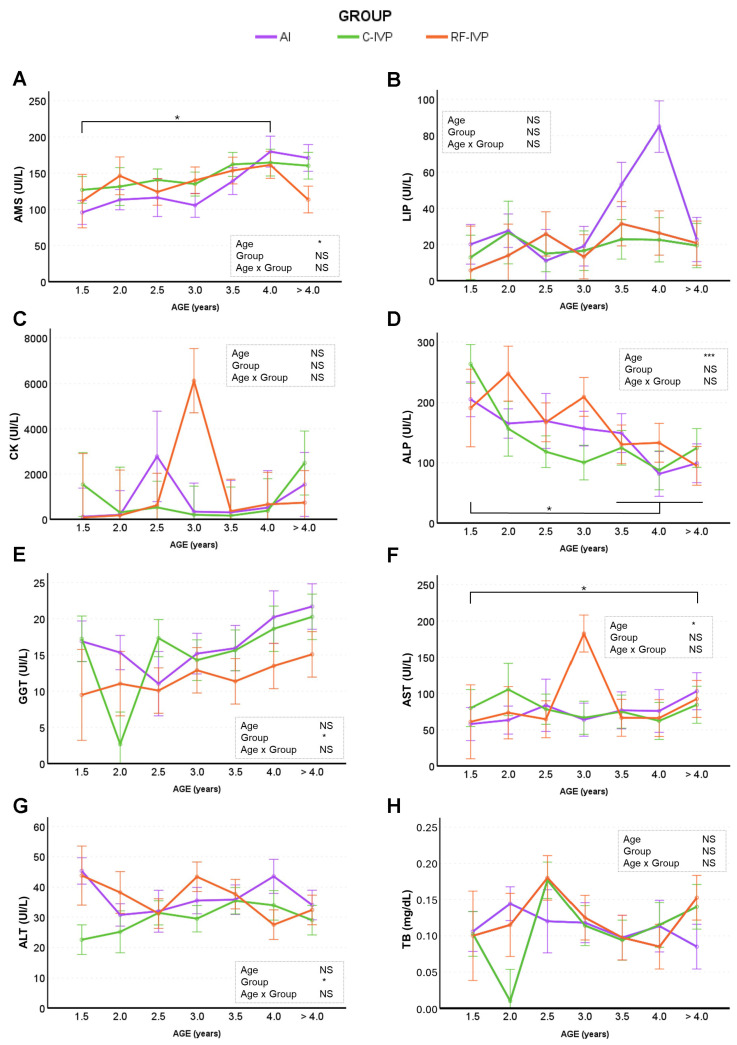
Biochemical parameters (hepatic, biliary, and gastrointestinal biomarkers) across age obtained in cattle born by artificial insemination (AI) and transfer of in vitro embryo produced under a standard protocol (C-IVP), and including reproductive fluids (RF-IVP). Data are expressed as mean ± SEM. (**A**) Amylase (AMS; UI/L), (**B**) lipase (LIP; UI/L), (**C**) creatinine kinase (CK; UI/L), (**D**) alkaline phosphatase (ALP; UI/L), (**E**) gamma-glutamyl transpeptidase (GGT; UI/L), (**F**) aspartate aminotransferase (AST; UI/L), (**G**) alanine aminotransferase (ALT; UI/L), and (**H**) total bilirubin (TB; mg/dL). Asterisk shows statistical difference at * *p* < 0.05, and *** *p* < 0.001; NS, not significant. Horizontal lines denote that all encompassed time points are significantly different. When the vertical lines are added, only the marked time points are significantly different.

The analysis of the biochemical profile showed that certain parameters, including triglycerides (TRIG), lipase (LIP), creatine kinase (CK), and total bilirubin (TB), were not influenced by group or age (Figure 5 and Figure 6). However, some other biochemical parameters showed significant differences between groups, such as creatinine (CREA; Figure 5D) and cholesterol (CHOL; Figure 5G), which were lower in the AI group compared with the C-IVP and RF-IVP groups. Gamma-glutamyl transferase (GGT; Figure 6E) levels were lower in RF-IVP animals compared to C-IVP and AI groups, whereas alanine aminotransferase (ALT; Figure 6E) levels were lower in C-IVP animals compared to AI and RF-IVP. The evaluation of age-related effects on biochemical parameters revealed significant differences in total protein (TP), albumin (ALB), globulin (GLOB), creatinine (CREA), urea, glucose (GLUC), amylase (AMS), alkaline phosphatase (ALP), and aspartate aminotransferase (AST) (Figure 5 and Figure 6). Total protein levels (Figure 5A) increased progressively with age, being lower in younger animals and significantly higher in those aged 4 years and older. Albumin levels (Figure 5B) remained relatively stable from 1.5 to 4 years but decreased significantly after 4 years. In contrast, globulin levels increased with age, rising steadily from 1.5 years to a peak in animals older than 4 years (Figure 5C). Creatinine (Figure 5D) and urea levels (Figure 5E) also showed an age-related increase, with older animals having higher levels than younger ones. Glucose levels (Figure 5F) generally decreased with age but showed a transient increase at 3 and 3.5 years. Animals at younger ages (1.5 years) exhibited higher glucose levels than when they were older (>4 years), while levels at 3.5 years were higher than at 2 and 4 years. In summary, TP, GLOB, CREA, urea, AMS, and AST levels increased with age, whereas ALB, GLUC, and ALP levels tended to decrease. Mean values for ALB, CHOLES, TRIG, and CK were higher in females than males. A significant age–sex interaction was observed for CHOLES and TRIG.

## 4. Discussion

Interpreting physical and laboratory results from large animals is challenging owing to a lack of detailed reference ranges by age, sex, season, and breed. Moreover, there is conflicting information on growth in animals of different embryo origins. We found that the average BMI values did not differ between the groups, but the BMI of all animals increased with age, which is consistent with previous results on the same herd [18] and other studies showing no differences in weight at birth, weaning, and at 1 year of age in cattle with different embryo origins [18,24,25,26].

Similar to the present study, no differences in body temperature have been reported between animals born after different reproductive techniques [18,24]. The observed highest temperature values at the youngest age and the decrease during late adulthood are reported here for the first time in cattle but they have been already reported in mice and humans [27,28,29,30,31], attributed to a reduction in metabolism leading to lower heat production and therefore a decrease in body temperature [32]. The hypothesis that a decrease in body temperature occurs with age and is caused by a decrease in metabolic activity is supported by the age-related decrease in ALP levels (this study) and thyroxine concentration observed in this herd [22]. However, body temperature variations might be due to external seasonal temperature variations [33]. Nevertheless, this hypothesis is rejected since body temperature was recorded during the warm periods of autumn and summer.

In agreement with other authors [24,34] and a previous study on the same herd [18], heart and respiratory rates were similar between groups. Despite the lack of data on older cattle, the observed decrease in respiratory and heart rates with age is consistent with observations in humans [35]. The higher heart and respiratory rates observed during the first three years of life, compared to older ages, may be due to a decrease in activity and stress levels during sampling [36], as the animals become accustomed to handling and restraint during sampling over time.

Previous studies in cattle have shown haematological changes with age, primarily in young animals [37,38], but there is no information on how these changes are maintained throughout life. During the first year of life of this herd, there were no differences in RBC values between groups, but an age effect was reported [18]. The current study confirms that these differences persist throughout life with an increase in MCV, MCH, MCHC, CH, and CHDW values and a decrease in RBC, RDW, and HDW. The erythrocyte values obtained were within the normal range when compared with the limited data available on three- to six-year-old cows [39]. As for the leucogram, the number of WBC and lymphocytes tended to decrease with age, which is the opposite of what happened during the first year of life [18]. This is consistent with the findings of Kramer [37], who reported higher WBC counts in three-year-old cattle than in older animals. The observed decrease in WBC and lymphocytes, and the increase in neutrophils, throughout life can be explained by immune system dysregulation with age. Similar observations have been reported in calves [40] and humans [41] due to immunosenescence. The differences between groups in basophil concentration observed in early life [18] were maintained throughout life. Regarding platelet information, it is well known that the platelet count and mean platelet volume show opposite patterns, since in the presence of a low platelet count, thrombopoietin stimulates megakaryocytes in the bone marrow to produce platelets [42], which could explain the observed values in the C-IVP group at two years of age.

The lack of studies and reference values for older cattle (≥2–3 years old) and from IVP programs makes it difficult to compare the biochemical data. Although variations were observed in many parameters, all of the obtained values were within the reference intervals for adult dairy and beef cattle [21]. These changes in biochemical values may indicate greater heterogeneity in IVP calves than in calves born by AI, as previously reported [43,44]. Cholesterol and creatinine values were lower in the AI group than in the IVP groups, consistent with observations made during the first year of life in this herd [18]. Alongside the observed increase in urea in older animals, the differences in renal function biomarkers between the AI and IVP groups could reflect changes in kidney function with age rather than a true effect of embryonic origin. Indeed, a decrease in renal function correlates with an increase in amylase [45], as was observed in all groups in this study. Despite the variations, the renal parameter values were within the adult reference ranges [21]. Elevated levels of alkaline phosphatase have been reported to be associated with liver and skeletal issues, and the concentration of this enzyme decreases with age [44]. Here, the activity of alkaline phosphatase decreased with age, in a manner similar to that observed during the early growth of animals [18]. Liver function is also correlated with alanine aminotransferase, gamma-glutamyl transpeptidase, and aspartate aminotransferase activities, and as expected, in this study, alanine aminotransferase remained stable throughout life and aspartate aminotransferase increased with age within normal ranges in adult cattle [21]. Further studies involving larger numbers of animals would be necessary to confirm whether the lower levels of gamma-glutamyl transpeptidase in RF-IVP and alanine aminotransferase in C-IVP, as compared to AI animals, might indicate different liver and skeletal functions that are clinically relevant. Sex-dependent differences in haematological and biochemical parameters were observed. Previous studies in dairy calves showed no effect of sex on haematology and serum biochemistry from birth to three months of age [38]. We did not observe an effect of sex in the same colony of animals up to 1 year of age [18] but hematic differences between males and females have been reported in other cattle studies [46] and are considered a normal finding.

## 5. Conclusions

In conclusion, this observational study provides the first reference values for physical, haematological, and biochemical parameters in cattle derived from different ARTs throughout life. Despite the differences found between groups, age-related variations were more pronounced. All parameters remained within normal ranges, demonstrating that IVP supports the production of healthy cattle, as does the AI method. This information is essential to improve the health management of in vitro-produced animals.

## Data Availability

The dataset is available upon request from the authors.

## Supplementary Materials

The following supporting information can be downloaded at https://www.mdpi.com/article/10.3390/ani15121763/s1: Table S1: Mean values of physical parameters sorted by age; Table S2: Mean values of haematological parameters (red blood cells) sorted by age; Table S3: Mean values of haematological parameters (white blood cells) sorted by age; Table S4: Mean values of haematological parameters (platelets and reticulocytes) sorted by age; Table S5: Mean values of biochemical parameters (protein, glycaemic, and kidney biomarkers) sorted by age; Table S6: Mean values of biochemical parameters (hepatic, biliary, and gastrointestinal biomarkers) sorted by age.

## Abbreviations

The following abbreviations are used in this manuscript:

ALB	Albumin
ALT	Alanine aminotransferase
ALP	Alkaline phosphatase
AMS	Amylase
AST	Aspartate aminotransferase
BMI	Body mass index
CHr	Reticulocyte haemoglobin content
CHCM	Cell haemoglobin concentration mean
CHDW	Cell haemoglobin distribution width
CHOL	Cholesterol
CK	Creatinine kinase
CREA	Creatinine
GGT	Gamma-glutamyl transpeptidase
GLOB	Globulin
GLUC	Glucose
HDW	Haemoglobin distribution width
MCH	Mean corpuscular haemoglobin
MCHC	Mean cell haemoglobin concentration
MCV	Mean corpuscular volume
MCVr	Mean corpuscular volume of reticulocyte
LIP	Lipase
MPV	Mean platelet volume
MPC	Mean platelet component
MPM	Mean platelet mass
PCT	Plateletcrit
PDW	Platelet distribution width
PLT	Platelets
RBC	Red blood cells
RDW	Red blood cell distribution width
TB	Total bilirubin
TP	Total protein
TRIG	Triglycerides
WBC	White blood cells
