# Supplementary material for: Long-Term Study of Physical, Haematological, and Biochemical Parameters in Cattle with Different Embryo Origins
Source: Animals (Basel). 2025 Jun 14;15(12):1763. doi: 10.3390/ani15121763 (PMC12189840; doi:10.3390/ani15121763)
Supplement: Supplementary file 1 [file animals-15-01763-s001.zip › animals-3665869-supplementary.pdf]

Supplementary tables

Supplementary Table S1. Mean values of physical parameters sorted by age. BMI (body mass index; Kg/m^2), respiratory rate (RPM, breaths per minute).

**Age (years)**		**BMI (Kg/m^2)**	**Body Temperature (˚C)**	**RPM (breaths/minute)**	**Heart rate (beats/minute)**
1.5	*n*	12	12	12	12
	Mean	18.95	37.70	36.00	64.50
	SEM	1.05	0.65	8.00	4.50
	Min	17.91	37.05	28.00	60.00
	Max	20.01	38.35	44.00	69.00
	SD	1,49	0,92	11,31	6,36
2	*n*	11	11	11	11
	Mean	17.61	38.94	38.18	52.91
	SEM	0.42	0.09	3.02	3.00
	Min	15.14	38.50	28.00	42.00
	Max	19.47	39.30	60.00	72.00
	SD	1.40	0.30	10.02	9.97
2.5	*n*	13	13	13	13
	Mean	19.10	38.43	38.92	67.46
	SEM	0.44	0.16	3.42	4.88
	Min	17.03	37.20	22.00	44.00
	Max	22.29	39.20	64.00	92.00
	SD	1.60	0.59	12.32	17.61
3	*n*	14	14	14	14
	Mean	19.73	38.06	36.00	78.86
	SEM	0.34	0.18	2.84	5.18
	Min	18.16	37.00	16.00	56.00
	Max	22.98	39.40	52.00	112.00

	SD	1.26	0.66	10.64	19.37
3.5	*n*	13	13	13	13
	Mean	21.29	38.52	28.00	80.31
	SEM	1.27	0.11	1.69	4.97
	Min	16.58	37.90	20.00	64.00
	Max	34.67	39.00	40.00	112.00
	SD	4.58	0.39	6.11	17.92
4	*n*	10	11	11	11
	Mean	21.56	38.36	24.36	77.45
	SEM	1.48	0.21	1.92	3.90
	Min	17.76	37.50	16.00	60.00
	Max	33.38	39.40	32.00	108.00
	SD	4.68	0.71	6.38	12.93
> 4.0	*n*	11	11	11	11
	Mean	21.09	38.41	26.36	70.45
	SEM	0.69	0.14	3.23	2.65
	Min	17.00	37.60	16.00	58.00
	Max	25.66	39.00	56.00	84.00
	SD	2.29	0.45	10.72	8.79
All	*n*	84	85	85	85
	Mean	20.01	38.42	32.32	71.51
	SEM	0.36	0.07	1.27	2.00
	Min	15.14	37.00	16.00	42.00
	Max	34.67	39.40	64.00	112.00
	SD	3.12	0.60	10.98	17.33

Supplementary Table S2. Mean values of hematological parameters (red blood cells) sorted by age. Haematocrit (%), Erythrocytes (x10^6 cells/µL), Haemoglobin (g/dL), mean corpuscular volume (MCV; fL), mean corpuscular hemoglobin (MCH; pg), mean corpuscular hemoglobin concentration (MCHC; g/dL), cell hemoglobin concentration mean (CHCM (g/dL), red blood cells distribution width (RDW, %), hemoglobin content (CH; pg), cell hemoglobin distribution width (CHDW; pg) and hemoglobin distribution width (HDW ;g/dL).

**Age (years)**		**Haematocrit (%)**	**Erythrocytes (x10^6 cells/µL)**	**Haemoglobin (g/dL)**	**MCV (fL)**	**MCH (pg)**	**MCHC (g/dL)**	**CHCM (g/dL)**	**RDW (%)**	**CH (pg)**	**CHDW (pg)**	**HDW (g/dL)**
1.5	*n*	9	9	9	9	9	9	9	9	9	9	9
	Mean	34.42	9.13	12.70	37.93	14.00	36.84	36.96	18.67	13.96	2.18	2.37
	SEM	0.67	0.32	0.27	0.95	0.38	0.23	0.22	0.42	0.37	0.09	0.04
	Min	31.40	7.59	11.30	33.80	12.20	35.90	35.90	16.70	12.20	1.83	2.19
	Max	37.70	10.43	13.70	42.00	15.90	37.80	37.90	20.80	15.60	2.73	2.56
	SD	2.02	0.95	0.80	2.84	1.14	0.69	0.67	1.26	1.10	0.26	0.13
2	*n*	11	11	11	11	11	11	11	11	11	11	11
	Mean	30.79	7.56	11.78	40.66	15.55	38.26	36.36	16.90	14.77	2.16	2.20
	SEM	1.06	0.12	0.41	0.94	0.38	0.34	0.25	0.46	0.31	0.04	0.05
	Min	25.60	6.77	9.50	35.60	13.30	37.00	35.00	15.50	12.90	1.95	2.02
	Max	36.80	8.36	13.60	44.00	17.30	41.00	37.70	20.10	15.90	2.36	2.48
	SD	3.50	0.41	1.37	3.10	1.27	1.13	0.83	1.52	1.03	0.12	0.15
2.5	*n*	13	13	13	13	13	13	13	13	13	13	13
	Mean	31.81	7.73	12.13	41.17	15.69	38.10	36.40	16.92	14.97	2.19	2.25
	SEM	1.03	0.21	0.42	0.76	0.31	0.20	0.27	0.21	0.28	0.05	0.04
	Min	25.80	6.21	9.60	35.60	13.90	37.10	35.00	15.70	13.60	1.87	2.02
	Max	37.60	8.75	14.50	45.00	17.70	39.80	38.20	18.70	16.80	2.44	2.53
	SD	3.70	0.75	1.52	2.73	1.13	0.72	0.96	0.76	1.01	0.18	0.15
3	*n*	15	15	15	15	15	15	15	15	15	15	15
	Mean	32.97	8.06	12.48	40.79	15.43	37.78	36.40	17.30	14.81	2.21	2.24
	SEM	1.34	0.22	0.55	0.79	0.36	0.26	0.19	0.22	0.30	0.05	0.03
	Min	24.10	6.12	8.80	35.20	13.10	36.20	34.90	16.10	12.90	1.85	2.01
	Max	41.30	9.71	15.70	45.10	17.50	39.40	37.60	18.80	16.50	2.47	2.47

	SD	5.18	0.84	2.12	3.07	1.39	1.01	0.74	0.86	1.14	0.20	0.12
3.5	*n*	13	13	13	13	13	13	13	13	13	13	13
	Mean	33.45	8.13	12.49	41.16	15.39	37.35	35.88	17.98	14.76	2.29	2.12
	SEM	0.92	0.15	0.34	0.90	0.34	0.16	0.23	0.37	0.34	0.06	0.03
	Min	28.60	7.48	10.60	36.80	13.60	36.70	34.90	15.30	13.20	1.94	1.94
	Max	39.40	9.22	14.50	45.80	17.20	38.40	37.90	20.60	16.60	2.70	2.32
	SD	3.30	0.55	1.22	3.23	1.24	0.56	0.82	1.33	1.22	0.20	0.10
4	*n*	11	11	11	11	11	11	11	11	11	11	11
	Mean	33.30	7.73	12.51	43.01	16.12	37.47	36.73	17.45	15.79	2.40	2.22
	SEM	1.63	0.33	0.66	0.71	0.31	0.27	0.16	0.29	0.27	0.06	0.04
	Min	25.70	6.47	9.40	39.80	14.50	36.50	36.10	15.50	14.40	2.16	2.05
	Max	44.10	9.85	16.70	47.10	17.80	39.40	38.00	18.70	17.40	2.78	2.40
	SD	5.40	1.09	2.20	2.36	1.03	0.88	0.54	0.96	0.91	0.19	0.12
> 4.0	*n*	12	12	12	12	12	12	12	12	12	12	12
	Mean	35.68	7.97	13.22	44.77	16.60	37.01	36.21	17.68	16.20	2.50	2.12
	SEM	1.19	0.18	0.45	0.91	0.39	0.21	0.12	0.40	0.33	0.06	0.04
	Min	31.40	7.23	11.30	39.80	14.30	35.90	35.30	16.10	14.40	2.22	1.93
	Max	43.40	9.26	16.00	50.40	18.80	38.50	36.70	20.60	18.00	2.94	2.29
	SD	4.14	0.63	1.55	3.15	1.35	0.72	0.42	1.37	1.13	0.20	0.13
All	*n*	84	84	84	84	84	84	84	84	84	84	84
	Mean	33.16	8.01	12.47	41.44	15.58	37.57	36.39	17.51	15.06	2.28	2.21
	SEM	0.46	0.09	0.18	0.37	0.15	0.10	0.08	0.14	0.13	0.02	0.02
	Min	24.10	6.12	8.80	33.80	12.20	35.90	34.90	15.30	12.20	1.83	1.93
	Max	44.10	10.43	16.70	50.40	18.80	41.00	38.20	20.80	18.00	2.94	2.56
	SD	4.23	0.86	1.63	3.40	1.38	0.94	0.78	1.24	1.24	0.22	0.15

Supplementary Table S3. Mean values of hematological parameters (white blood cells) sorted by age.

Age (years)		WBC ($x10^3$ cells/µL)	Neutrophils (%)	Neutrophils ($x10^3$ cells/µL)	Lymphocytes (%)	Lymphocytes ($x10^3$ cells/µL)	Monocytes (%)	Monocytes ($x10^3$ cells/µL)	Eosinophils (%)	Eosinophils ($x10^3$ cells/µL)	Basophils (%)	Basophils ($x10^3$ cells/µL)
1.5	*n*	9	9	9	9	9	9	9	9	9	9	9
	Mean	7.31	26.51	1.93	65.43	4.78	2.26	0.16	4.14	0.31	1.09	0.08
	SEM	0.37	1.57	0.15	1.42	0.27	0.24	0.02	0.31	0.03	0.08	0.01
	Min	5.61	20.80	1.38	58.50	3.47	1.40	0.11	2.90	0.16	0.80	0.05
	Max	8.60	32.80	2.74	69.90	6.01	3.50	0.26	5.80	0.41	1.40	0.11
	SD	1.12	4.71	0.46	4.26	0.82	0.71	0.05	0.94	0.09	0.23	0.02
2	*n*	11	11	11	11	11	11	11	11	11	11	11
	Mean	5.25	27.06	1.54	60.96	3.10	3.29	0.18	5.46	0.25	3.39	0.17
	SEM	0.64	2.12	0.26	1.99	0.34	0.48	0.03	0.90	0.03	0.50	0.03
	Min	1.51	13.00	0.20	50.10	1.05	0.70	0.01	2.40	0.11	1.70	0.05
	Max	8.13	37.60	2.85	72.30	5.02	5.80	0.34	11.90	0.48	8.00	0.32
	SD	2.13	7.02	0.85	6.60	1.14	1.60	0.10	2.98	0.12	1.65	0.09
2.5	*n*	13	13	13	13	13	13	13	13	13	13	13
	Mean	5.19	43.74	2.25	46.22	2.43	2.27	0.12	5.45	0.27	1.95	0.10
	SEM	0.41	5.20	0.29	4.58	0.34	0.30	0.02	0.96	0.04	0.33	0.02
	Min	3.19	24.50	1.01	16.30	0.79	0.20	0.01	1.30	0.05	0.50	0.02
	Max	8.56	78.70	3.94	65.60	4.79	3.70	0.25	13.20	0.52	4.10	0.27
	SD	1.48	18.75	1.06	16.51	1.24	1.09	0.07	3.46	0.14	1.20	0.07
3	*n*	15	15	15	15	15	15	15	15	15	15	15
	Mean	5.33	45.89	2.49	43.94	2.32	3.03	0.15	5.79	0.30	0.99	0.05
	SEM	0.33	3.84	0.33	3.81	0.28	0.62	0.03	0.68	0.03	0.08	0.00
	Min	3.49	27.80	1.34	4.80	0.36	0.70	0.05	3.10	0.15	0.50	0.02
	Max	7.40	85.40	6.32	64.70	4.28	10.10	0.44	11.70	0.62	1.50	0.10
	SD	1.29	14.87	1.27	14.77	1.08	2.39	0.11	2.63	0.12	0.32	0.02
3.5	*n*	13	13	13	13	13	13	13	13	13	13	13

	Mean	4.71	41.51	1.98	45.62	2.14	6.38	0.28	4.85	0.24	0.99	0.05
	SEM	0.37	3.84	0.23	4.24	0.23	0.92	0.03	0.65	0.04	0.14	0.01
	Min	2.46	13.30	0.51	20.10	0.49	1.10	0.04	2.10	0.06	0.40	0.01
	Max	6.90	63.40	2.97	82.40	3.66	13.10	0.41	10.90	0.64	2.20	0.13
	SD	1.34	13.86	0.82	15.27	0.81	3.30	0.11	2.35	0.16	0.50	0.03
4	*n*	11	11	11	11	11	11	11	11	11	11	11
	Mean	4.49	41.05	1.90	47.96	2.09	4.25	0.19	5.64	0.25	0.92	0.04
	SEM	0.40	4.12	0.25	5.29	0.28	0.69	0.03	1.50	0.06	0.12	0.01
	Min	1.92	14.40	0.28	19.00	1.03	0.60	0.01	1.40	0.03	0.40	0.02
	Max	6.68	66.50	3.70	82.10	3.85	7.70	0.41	20.20	0.74	1.70	0.07
	SD	1.32	13.66	0.84	17.54	0.93	2.30	0.11	4.99	0.19	0.39	0.02
> 4.0	*n*	12	12	12	12	12	12	12	12	12	12	12
	Mean	5.17	40.19	2.04	48.26	2.53	3.64	0.19	6.74	0.35	0.96	0.05
	SEM	0.31	2.55	0.14	3.26	0.26	0.56	0.03	0.69	0.04	0.13	0.01
	Min	3.70	27.80	1.52	23.20	1.03	1.50	0.06	3.00	0.12	0.40	0.02
	Max	6.82	58.30	3.28	61.30	4.13	7.90	0.35	11.30	0.60	1.90	0.08
	SD	1.06	8.83	0.49	11.29	0.91	1.95	0.10	2.38	0.16	0.44	0.02
All	*n*	84	84	84	84	84	84	84	84	84	84	84
	Mean	5.28	38.89	2.05	50.23	2.68	3.63	0.18	5.49	0.28	1.45	0.07
	SEM	0.17	1.57	0.10	1.64	0.14	0.27	0.01	0.33	0.02	0.13	0.01
	Min	1.51	13.00	0.20	4.80	0.36	0.20	0.01	1.30	0.03	0.40	0.01
	Max	8.60	85.40	6.32	82.40	6.01	13.10	0.44	20.20	0.74	8.00	0.32
	SD	1.57	14.37	0.92	15.02	1.26	2.45	0.10	3.02	0.14	1.16	0.06

Supplementary Table S4. Mean values of hematological parameters (platelets and reticulocytes) sorted by age. Platelets (PLT; x103; cells/µL), mean platelet volume (MPV; fL), plateletcrit (PCT; %), platelet distribution width (PDW; %), mean platelet content (MPC), plateletcrit distribution width (PCDW), mean platelet mass (MPM), platelet mass distribution width (PMDW), proportion of large platelets, reticulocyte hemoglobin content (CHr), and reticulocytes mean corpuscular volume (MCVr).

**Age (years)**		**PLT ($x10^3$ cells/µL)**	**MPV (fL)**	**PCT (%)**	**PDW (%)**	**MPC (g/dL)**	**PCDW (g/dL)**	**MPM (pg)**	**PMDW (pg)**	**Large PLT ($x10^3$ cells/µL)**	**Reticulocytes (%)**	**Reticulocytes (10^3 cell/µL)**	**CHr (pg)**	**MCV r(fL)**
1.5	*n*	9	9	9	9	9	9	9	9	9	9	9	9	9
	Mean	189.67	6.98	0.13	80.99	26.50	7.24	1.57	0.77	5.44	0.05	4.56	17.23	53.90
	SEM	25.58	0.38	0.01	3.34	0.63	0.25	0.04	0.03	1.48	0.01	0.60	0.68	2.64
	Min	99.00	5.20	0.08	67.40	24.10	6.40	1.32	0.68	1.00	0.03	2.00	13.50	39.70
	Max	324.00	8.80	0.19	100.80	30.40	8.60	1.72	0.93	16.00	0.09	7.00	20.30	65.00
	SD	76.75	1.13	0.04	10.01	1.89	0.74	0.13	0.08	4.45	0.02	1.81	2.03	7.92
2	*n*	11	11	11	11	11	11	11	11	11	11	11	11	11
	Mean	142.00	10.27	0.12	83.15	24.26	7.68	1.68	0.84	11.64	0.06	4.82	15.81	46.59
	SEM	22.82	2.04	0.01	4.27	1.97	0.56	0.09	0.04	4.45	0.01	1.11	0.57	1.84
	Min	66.00	5.30	0.07	54.80	10.60	4.40	1.36	0.64	1.00	0.02	1.00	12.50	37.50
	Max	285.00	25.80	0.19	104.30	29.70	10.50	2.36	1.13	47.00	0.15	12.00	18.80	54.20
	SD	75.69	6.77	0.04	14.15	6.54	1.84	0.30	0.15	14.75	0.05	3.68	1.88	6.09
2.5	*n*	13	13	13	13	13	13	13	13	13	13	13	13	13
	Mean	170.92	6.76	0.12	83.12	27.18	7.16	1.55	0.76	4.85	0.06	4.38	15.77	47.10
	SEM	9.32	0.38	0.01	2.95	0.49	0.24	0.03	0.02	1.96	0.01	0.70	0.36	1.36
	Min	114.00	5.60	0.07	69.30	23.60	6.00	1.42	0.69	1.00	0.02	1.00	13.60	39.20
	Max	238.00	10.90	0.20	104.10	30.80	9.80	1.75	0.96	28.00	0.14	11.00	18.10	56.40
	SD	33.61	1.35	0.04	10.64	1.77	0.86	0.10	0.08	7.07	0.03	2.53	1.29	4.89
3	*n*	15	15	15	15	15	15	15	15	15	15	15	15	15
	Mean	224.33	7.43	0.16	77.03	25.89	7.33	1.60	0.76	6.87	0.09	7.07	15.22	44.48
	SEM	18.40	0.44	0.01	2.59	0.68	0.21	0.04	0.02	1.80	0.01	1.07	0.38	1.35
	Min	111.00	5.60	0.10	65.40	18.80	6.30	1.36	0.65	1.00	0.02	1.00	12.80	34.40
	Max	366.00	12.20	0.25	96.80	29.10	9.70	1.86	0.89	23.00	0.14	13.00	18.50	54.50

	SD	71.25	1.69	0.04	10.05	2.63	0.81	0.14	0.08	6.96	0.05	4.15	1.48	5.23
3.5	*n*	13	13	13	13	13	13	13	13	13	13	13	13	13
	Mean	238.85	7.77	0.17	77.66	25.21	7.05	1.62	0.78	6.31	0.04	3.15	15.75	47.22
	SEM	27.05	0.59	0.01	2.06	0.71	0.26	0.04	0.03	1.00	0.00	0.37	0.69	2.79
	Min	66.00	6.50	0.10	65.60	18.80	6.20	1.38	0.63	3.00	0.02	2.00	11.50	32.10
	Max	483.00	14.60	0.31	92.50	28.70	9.70	1.87	0.97	17.00	0.07	6.00	19.30	63.70
	SD	97.55	2.12	0.05	7.44	2.57	0.92	0.14	0.10	3.61	0.02	1.34	2.48	10.05
4	*n*	11	11	11	11	11	11	11	11	11	11	11	11	11
	Mean	210.82	8.70	0.16	75.46	24.87	7.30	1.67	0.77	7.45	0.07	5.27	17.25	51.54
	SEM	23.17	1.60	0.01	2.17	1.25	0.13	0.09	0.04	2.71	0.02	1.43	0.84	3.34
	Min	61.00	6.20	0.12	62.20	13.40	6.80	1.45	0.64	3.00	0.01	1.00	13.10	35.00
	Max	343.00	24.50	0.23	85.60	27.90	8.40	2.50	1.19	34.00	0.20	18.00	22.20	72.40
	SD	76.83	5.32	0.03	7.20	4.13	0.42	0.29	0.15	8.98	0.05	4.73	2.79	11.07
> 4.0	*n*	12	12	12	12	12	12	12	12	12	12	12	12	12
	Mean	139.58	8.55	0.12	81.30	24.82	7.26	1.74	0.82	5.58	0.04	3.50	17.47	53.84
	SEM	13.36	0.41	0.01	2.46	0.52	0.22	0.04	0.03	0.91	0.01	0.58	0.68	3.06
	Min	62.00	7.00	0.06	67.60	21.00	6.10	1.53	0.69	3.00	0.01	1.00	14.50	40.30
	Max	209.00	11.10	0.15	94.20	26.80	8.30	2.00	0.96	12.00	0.09	8.00	21.50	72.60
	SD	46.30	1.44	0.03	8.54	1.81	0.76	0.15	0.09	3.15	0.02	2.02	2.36	10.60
All	*n*	84	84	84	84	84	84	84	84	84	84	84	84	84
	Mean	189.94	8.03	0.14	79.70	25.55	7.28	1.63	0.79	6.83	0.06	4.74	16.27	48.86
	SEM	8.47	0.38	0.00	1.09	0.37	0.11	0.02	0.01	0.85	0.00	0.36	0.24	0.95
	Min	61.00	5.20	0.06	54.80	10.60	4.40	1.32	0.63	1.00	0.01	1.00	11.50	32.10
	Max	483.00	25.80	0.31	104.30	30.80	10.50	2.50	1.19	47.00	0.20	18.00	22.20	72.60
	SD	77.60	3.46	0.05	9.97	3.39	0.96	0.19	0.11	7.79	0.04	3.31	2.18	8.67

Supplementary Table S5. Mean values of biochemical parameters (protein, glycemic and kidney biomarkers) sorted by age. Total proteins (TP; g/dL), albumin (ALB; g/dL), globulin (GLOB; g/dL), creatinine (CREA; mg/dL), glucose (GLUC; mg/dL), (G) cholesterol (CHOL; mg/dL) and triglycerides (TRIG; mg/dL).

AGE (years)		PROT (g/dl)	ALB (g/dl)	GLOB (g/dl)	ALB/GLOB ratio	CREA (mg/dl)	UREA (mg/dl)	GLUC (mg/dl)	CHOL (mg/dl)	TRIGL (mg/dl)
1.5	*n*	10	10	10	10	10	10	10	10	10
	Mean	6.11	3.49	2.61	1.47	1.76	22.23	70.59	118.36	15.36
	SEM	0.20	0.07	0.25	0.17	0.11	1.96	2.64	10.76	1.57
	Min	5.14	3.04	1.53	0.73	1.17	12.80	62.70	69.73	9.26
	Max	7.18	3.79	4.14	2.45	2.46	33.60	85.60	188.48	22.79
	SD	0.64	0.23	0.78	0.53	0.36	6.21	8.36	34.04	4.96
2	*n*	11	11	11	11	11	11	11	11	11
	Mean	6.79	3.34	3.45	1.02	1.45	15.11	61.77	155.28	12.80
	SEM	0.20	0.09	0.27	0.08	0.06	1.42	1.79	12.14	1.66
	Min	6.13	2.61	2.62	0.44	1.15	8.90	52.60	100.43	7.36
	Max	8.54	3.85	5.93	1.35	1.75	22.10	73.00	224.46	26.55
	SD	0.65	0.30	0.89	0.25	0.19	4.71	5.94	40.28	5.51
2.5	*n*	12	12	12	12	12	12	12	12	12
	Mean	6.61	3.32	3.29	1.02	1.75	14.98	66.72	143.93	11.40
	SEM	0.15	0.06	0.12	0.04	0.09	1.99	2.95	10.62	1.41
	Min	5.58	2.93	2.34	0.86	1.26	5.50	51.30	100.28	5.90
	Max	7.29	3.61	3.78	1.38	2.27	28.50	84.30	229.96	21.77
	SD	0.52	0.20	0.42	0.15	0.30	6.90	10.21	36.77	4.88
3	*n*	14	14	14	14	14	14	14	14	14
	Mean	6.49	3.17	3.32	0.99	1.64	17.34	73.24	125.36	10.86
	SEM	0.14	0.07	0.15	0.06	0.08	2.78	6.20	8.60	0.76
	Min	5.66	2.88	2.47	0.67	1.25	4.00	45.60	92.19	5.48
	Max	7.80	3.87	4.66	1.57	2.26	41.20	141.50	193.14	15.23
	SD	0.53	0.26	0.57	0.22	0.29	10.40	23.20	32.19	2.84
3.5	*n*	13	13	13	13	13	13	13	13	13

	Mean	6.97	3.20	3.77	1.07	1.59	22.19	74.85	111.34	11.52
	SEM	0.38	0.05	0.41	0.19	0.08	3.27	2.23	8.36	1.48
	Min	4.78	2.91	1.20	0.51	1.11	8.90	61.20	69.86	2.82
	Max	8.61	3.58	5.70	2.98	2.12	43.70	91.70	153.39	20.92
	SD	1.38	0.19	1.46	0.69	0.30	11.77	8.04	30.14	5.35
4	*n*	11	11	11	11	11	11	11	11	11
	Mean	7.74	3.16	4.59	0.71	1.73	23.01	64.27	116.42	14.55
	SEM	0.18	0.09	0.22	0.05	0.15	2.83	2.10	12.04	1.33
	Min	6.56	2.38	3.45	0.40	1.19	12.00	52.20	66.37	7.73
	Max	8.73	3.50	5.89	0.90	2.50	39.20	74.60	197.85	20.85
	SD	0.59	0.29	0.73	0.15	0.50	9.38	6.98	39.95	4.40
> 4.0	*n*	12	12	12	12	12	12	12	12	12
	Mean	7.41	2.58	4.83	0.58	2.04	27.05	58.50	136.42	15.99
	SEM	0.10	0.26	0.28	0.09	0.16	1.97	1.61	10.58	2.35
	Min	6.86	1.40	3.69	0.22	1.40	12.70	46.50	93.11	6.10
	Max	8.12	3.80	6.65	0.99	3.26	40.60	68.30	205.64	28.94
	SD	0.36	0.91	0.97	0.29	0.54	6.81	5.57	36.65	8.15
All	*n*	83	83	83	83	83	83	83	83	83
	Mean	6.87	3.17	3.71	0.97	1.71	20.21	67.39	129.38	13.07
	SEM	0.10	0.05	0.12	0.05	0.04	1.02	1.41	4.10	0.60
	Min	4.78	1.40	1.20	0.22	1.11	4.00	45.60	66.37	2.82
	Max	8.73	3.87	6.65	2.98	3.26	43.70	141.50	229.96	28.94
	SD	0.88	0.48	1.11	0.44	0.39	9.25	12.80	37.39	5.49

Supplementary Table S6. Mean values of biochemical parameters (hepatic, biliary and gastrointestinal biomarkers) sorted by age. Amylase (AMS; UI/L), lipase (LIP; UI/L), creatinine kinase (CK; UI/L), alkaline phosphatase (ALP; UI/L), gamma-glutamyl transpeptidase (GGT; UI/L), aspartate aminotransferase (AST; UI/L), alanine aminotransferase (ALT; UI/L) and total bilirubin (TB; mg/dL).

AGE (years)		AMS (UI/L)	LIP (UI/L)	CK (UI/L)	ALP (UI/L)	GGT (UI/L)	AST (UI/L)	ALT (UI/L)	TB (mg/dl)
1.5	*n*	10	10	10	10	10	10	10	10
	Mean	109.86	15.86	687.06	227.18	16.30	67.25	36.09	0.10
	SEM	13.19	4.00	346.48	21.62	2.00	5.61	4.44	0.01
	Min	41.40	2.10	62.20	86.00	9.50	50.10	18.30	0.07
	Max	183.30	32.20	2904.30	333.80	31.90	103.70	56.20	0.13
	SD	41.72	12.64	1095.66	68.36	6.33	17.73	14.05	0.02
2	*n*	11	11	11	11	11	11	11	11
	Mean	122.75	25.04	221.11	178.62	12.26	73.09	31.11	0.11
	SEM	11.90	3.42	24.79	21.89	2.17	7.12	1.95	0.02
	Min	47.80	12.00	132.70	66.00	0.10	46.40	21.70	0.01
	Max	178.10	44.80	375.60	340.20	24.90	135.00	39.40	0.20
	SD	39.46	11.35	82.20	72.61	7.18	23.62	6.46	0.07
2.5	*n*	12	12	12	12	12	12	12	12
	Mean	131.19	18.01	940.69	143.18	13.88	74.88	31.48	0.17
	SEM	7.23	2.82	430.38	18.81	1.89	4.79	1.38	0.03
	Min	92.20	8.60	118.20	61.80	0.10	54.60	22.50	0.01
	Max	179.40	37.70	5355.00	293.60	25.40	107.10	39.80	0.41
	SD	25.05	9.77	1490.90	65.16	6.53	16.58	4.76	0.09
3	*n*	14	14	14	14	14	14	14	14
	Mean	126.01	16.59	1946.03	151.64	14.22	99.04	35.64	0.12
	SEM	11.62	2.70	1687.70	21.22	2.19	31.91	3.16	0.02
	Min	43.40	2.50	74.30	59.20	0.10	39.30	19.80	0.01
	Max	195.10	40.70	23880.00	370.80	26.30	508.80	57.80	0.17
	SD	43.49	10.09	6314.81	79.40	8.18	119.41	11.83	0.06
3.5	*n*	13	13	13	13	13	13	13	13

	Mean	152.48	34.92	274.26	134.14	14.43	73.16	36.25	0.10
	SEM	11.14	6.35	63.24	15.30	1.48	3.29	3.54	0.01
	Min	80.10	11.00	82.40	57.60	0.10	58.70	22.50	0.01
	Max	201.70	83.90	874.90	237.80	19.80	95.80	63.90	0.16
	SD	40.15	22.90	228.02	55.15	5.33	11.87	12.77	0.05
4	*n*	11	11	11	11	11	11	11	11
	Mean	167.60	41.06	526.65	102.60	17.20	67.61	34.24	0.10
	SEM	9.47	17.98	170.56	22.02	1.75	6.56	4.08	0.02
	Min	104.00	9.50	78.50	60.80	1.60	36.60	16.60	0.01
	Max	196.10	216.60	1567.60	308.20	21.60	106.30	59.50	0.20
	SD	31.40	59.62	565.68	73.03	5.81	21.74	13.52	0.06
> 4.0	*n*	12	12	12	12	12	12	12	12
	Mean	148.39	21.06	1594.74	106.32	19.03	93.63	31.84	0.13
	SEM	8.84	3.07	643.42	7.25	1.37	5.23	1.22	0.01
	Min	84.60	9.00	116.00	79.20	12.90	67.60	26.20	0.07
	Max	187.70	35.70	7480.00	170.80	29.60	133.00	39.00	0.23
	SD	30.64	10.64	2228.89	25.12	4.74	18.13	4.21	0.05
All	*n*	83	83	83	83	83	83	83	83
	Mean	137.28	24.59	919.65	147.30	15.28	79.28	33.85	0.12
	SEM	4.35	2.84	307.95	8.04	0.72	5.67	1.13	0.01
	Min	41.40	2.10	62.20	57.60	0.10	36.60	16.60	0.01
	Max	201.70	216.60	23880.00	370.80	31.90	508.80	63.90	0.41
	SD	39.65	25.87	2805.56	73.22	6.54	51.66	10.25	0.06
